# Smart-aggregation imaging for single molecule localisation with SPAD cameras

**DOI:** 10.1038/srep37349

**Published:** 2016-11-23

**Authors:** Istvan Gyongy, Amy Davies, Neale A. W. Dutton, Rory R. Duncan, Colin Rickman, Robert K. Henderson, Paul A. Dalgarno

**Affiliations:** 1 The University of Edinburgh, Institute for Integrated Micro and Nano Systems, Edinburgh, UK; 2 Heriot-Watt University, Institute of Biological Chemistry, Biophysics and Bioengineering, Edinburgh, EH14 4AS, UK; 3 STMicroelectronics Imaging Division, 33 Pinkhill, Edinburgh EH12 7BF, UK

## Abstract

Single molecule localisation microscopy (SMLM) has become an essential part of the super-resolution toolbox for probing cellular structure and function. The rapid evolution of these techniques has outstripped detector development and faster, more sensitive cameras are required to further improve localisation certainty. Single-photon avalanche photodiode (SPAD) array cameras offer single-photon sensitivity, very high frame rates and zero readout noise, making them a potentially ideal detector for ultra-fast imaging and SMLM experiments. However, performance traditionally falls behind that of emCCD and sCMOS devices due to lower photon detection efficiency. Here we demonstrate, both experimentally and through simulations, that the sensitivity of a binary SPAD camera in SMLM experiments can be improved significantly by aggregating only frames containing signal, and that this leads to smaller datasets and competitive performance with that of existing detectors. The simulations also indicate that with predicted future advances in SPAD camera technology, SPAD devices will outperform existing scientific cameras when capturing fast temporal dynamics.

Single molecule localisation microscopy (SMLM) is becoming increasingly established as a benchmark imaging modality to probe sub cellular structure and molecular dynamics within the living cell^1^,^2^,^3^. Inherently dependent on molecular blinking, and a photon starved technique, SMLM is one of the key applications requiring fast, high sensitivity cameras and camera development has therefore underpinned practical application of SMLM. Electron multiplying CCDs (emCCD) and scientific CMOS (sCMOS) are the leading technologies typically employed, with both offering sub-electron read noise and high optical sensitivity. The high gain of emCCDs offers a leading advantage at extremely low light levels, whilst the sCMOS cameras higher frame rate and smaller pixel sizes have advantages in particle tracking and dynamic applications^4^,^5^. However, these technologies remain limited in terms of sensitivity and frame rate and a technological trend in image sensors has been the push to achieve more rapid imaging and true single photon sensitivity through increased signal gain and electronic noise reduction^6^. Three solid-state imaging technologies have attained sub-electron read noise that, combined with high optical sensitivity, have an inherent capability of single photon resolution: the emCCD, CMOS Single Photon Avalanche Diode (SPAD), and two CMOS image sensors (CIS) devices, namely the Pinned Avalanche Photo Diode (PAPD)^7^, and the pinned photo-diode (PPD)^8^. However, high noise floors, lack of true single photon statistics and no time resolved modalities limit the practical use of emCCDs, CIS and PAPD devices in single photon counting applications.

CMOS SPAD array technology offers a viable solution for true widefield single photon imaging. In a rapidly developing technological field, we have recently shown how a reduction of in-pixel circuitry employing analogue counting and binary memory circuits has led to decreased pixel sizes, increased fill factors and high frame rates of over >10 k frames per second (FPS), all with negligible readout noise contributed from the digital electronics^9^. Combined with quantum efficiencies of above 35% in the visible spectrum, SPAD arrays now provide a platform for ultra-fast imaging for the life sciences. In this paper we explore the application to SMLM, a particularly challenging imaging modality in terms of photon budget and imaging speed, and show how SPAD arrays offer significant improvements over existing emCCD and sCMOS technology.

SMLM, which has many variants, uses either photo-activatable or stochastically activated fluorescent markers that are activated in sparse subsets at a time. The resulting spatially distinct diffraction-limited point spread functions are individually identified and localised, (using some form of point spread function model, center of mass or equivalent) allowing high resolution images of localised emitters to be constructed over time. The technique has seen many developments, including multi-colour and 3D imaging^10^, but the challenge, from an imaging perspective, remains the same: to capture the millisecond molecule blinks with suitably high signal-to-noise ratio, so that their position may be estimated with the highest accuracy and precision.

In this context, a high frame rate camera, operating faster than the molecules stochastic blinking rate, potentially allows for the precise detection of the onset and duration of a single blink event. Compared to traditional emCCD or sCMOS imaging, that aggregate photon acquisitions in fixed time windows of normally several 10’s msecs, this provides extra precision in molecule identification, time-windowing and signal intensity thresholding so that background noise may be suppressed and the integrated signal maximised. However, if the camera runs at sub-millisecond exposures there will typically be very few photons from the molecule, preventing molecules from being localised on single frames. Oversampling the temporal behaviour allows for the intelligent summation of multiple sequential frames to regain signal intensity whilst maximizing frame rate, but in existing cameras the total readout noise increases with the square root of the number of summed frames, degrading the signal-to-noise ratio. This paper sets out a frame summing scheme, termed smart aggregation, that exploits the negligible read noise of SPAD cameras so that no additional noise is incurred. Furthermore a model is presented to simulate optimal detector performance for localisation microscopy, highlighting the key technological gains that, in addition to smart aggregation, are needed to further optimize the application of single photon array detectors.

## SMLM with a binary SPAD camera

**Figure 1:**
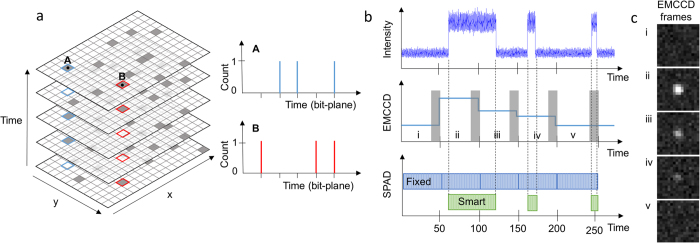
(**a**) Schematic representation of the output of a binary SPAD imager. Each pixel registers a 0 (no photon count, white) or 1 (photon count, grey) in time. (**b**) Representation of the operational frame capture of an emCCD and bit-plane imager for an example, single blinking molecule. The top graph represents the molecule intensity trace in time, the middle graph the collected intensity from a fixed frame rate emCCD. The grey regions represent data readout sectors. The bottom graph shows the difference between fixed frame capture and smart-aggregated frame capture for a SPAD CMOS camera. (**c**) Simulated point spread functions from the molecule emitter in each time frame as imaged in the emCCD.

The Quanta Image Sensor (QIS) concept^11^ projects the recent image sensor developments of read noise reduction, decreasing pixel size (and diminishing full well) to an imaging array of single photon photodetectors with a binary response. The binary states of either 0 (no photon detected) or 1 (at least one photon detected) provide limited information, therefore these are summed in space and/or time to form a spatio-temporally oversampled greyscale image frame. A binary SPAD camera is an example of a QIS, its raw output consisting of spatial information of binary bits or “bit-planes”, as illustrated in Fig. 1a, with each pixel producing a time-domain sequence of 1’s and 0’s.

A characteristic of such a SPAD camera is logarithmic compression in the response to light, akin to photographic film, due to the “pile-up” distortion of many incident photons recorded with the same logical high signal value as a single photon. This behaviour has been predicted by theory^12^, as well as verified experimentally using a SPAD imager^9^. Yet, in single molecule microscopy applications, the low light intensity and high frame rate, results in typically <0.2 photons/bit plane, and the SPAD camera would operate in the linear region of the response curve. Indeed, for low light applications, where one might have a few thousand incident photons/pixel/s, provided the frame rate is high enough (>1 k FPS), binary pixel values, when aggregated together, are sufficient to reproduce the variations in light intensity in a scene.

The main noise source in a SPAD device is the pixel dark count rate (DCR), which refers to the spurious firings of the SPAD due to thermal events. The level of DCR depends on the pixel size and architecture, and will vary across the pixel array (as quantified using the parameter Dark Rate Non Uniformity, or DRNU). In a similar way, there will be a Photon Response Non-Uniformity (PRNU) in the photon detection efficiency (PDE) of individual pixels that is the ratio between the average rate of photon detections and the rate of incident photons. Antolovic *et al*.^13^ discusses compensating for DCR, PRNU, and linearizing the response of SPAD QIS. Correcting for the DCR entails measuring the average count rate at each pixel, and subtracting the resulting “background” frame from subsequent images. The additional shot noise introduced by the dark count remains. Moreover, as in other camera technologies, SPAD imagers have a certain percentage of “hot pixels”, where the DCR is much higher than average and masks true photon detections, rendering the pixels unusable.

Crucial to the operation of a SPAD imager in QIS mode is the fact that there is negligible read-out noise, which means that an arbitrary number of bit-planes may be summed without incurring a noise penalty. This represents a distinct advantage in the imaging of blinking molecules in SMLM. Figure 1b illustrates the typical scenario when a stochastically blinking molecule is being imaged. The top graph shows the (simulated) light intensity trace from a single molecule (or multiple spatially indistinguishable molecules), comprising a longer and two shorter blinks of different durations due to the stochastic nature of the photophysics. If imaging with a conventional emCCD, integrating over fixed frames (i–v), produces peak intensity values shown in the middle graph, and the images shown in Fig. 1c. The long blink is captured mostly in frame ii, with a smaller proportion in frame iii. Frame iv shows a faint image of the short blink, due to the blink only appearing for a fraction of the integration period, and therefore being averaged out with the background. Importantly, frame v misses the final short blink entirely, as it aligns with the read-out period of that emCCD frame, a dead time in each frame where no data can be acquired. Figure 1b and c highlight the main challenges imaging single molecule blinking events with an emCCD. Frame ii and iii show a similar image, despite representing different blinking under different conditions, and events and information are missed in the read-out dead time of each frame. It is common in SMLM experiments to sum consecutive frames showing spatially coincident molecule emission, in an attempt to maximise signal to noise^2^. However, this approach cannot distinguish between one molecule that is on over multiple frames, and separate, spatially co-localised molecules which each blink on and off within single frames. A binary SPAD on the other hand camera captures bit-planes at a much faster rate than the blinking dynamics, without read-out dead time, to provide a temporal distinction between such events at rates orders of magnitudes above that of an emCCD. Each bit-planes could of course be summed over fixed periods to produce a similar output to an emCCD, but the approach proposed here is to sum only during the blink duration, Fig. 1c lowest panel. Noting that the lowest achievable localisation uncertainty (see, for example, ref. 14) is largely dependent on the ratio of detected molecule photons over background photons, the suggested “smart aggregation” approach will yield optimised localisation without loss of information.

The SPAD sensor explored here (labelled SPCImager) is a 320 × 240 resolution imager featuring an 8 μm pixel pitch at 26.8% fill factor, and a peak photon detection probability of 35% at 450 nm^15^. When operated in binary mode, bit-planes are captured at a rate of 10 kfps. Both rolling and global shutter modes are available, but the former is typically used as it allows for back-to-back exposures at the maximum frame rate (so that the exposure time per bit-plane is 100 μs). The imager is paired with an FPGA board (Opal Kelly XEM6310) that controls the acquisition of image data, relaying a continuous stream of bit-planes to a PC over a USB 3.0 link.

## Smart aggregation

**Figure 2:**
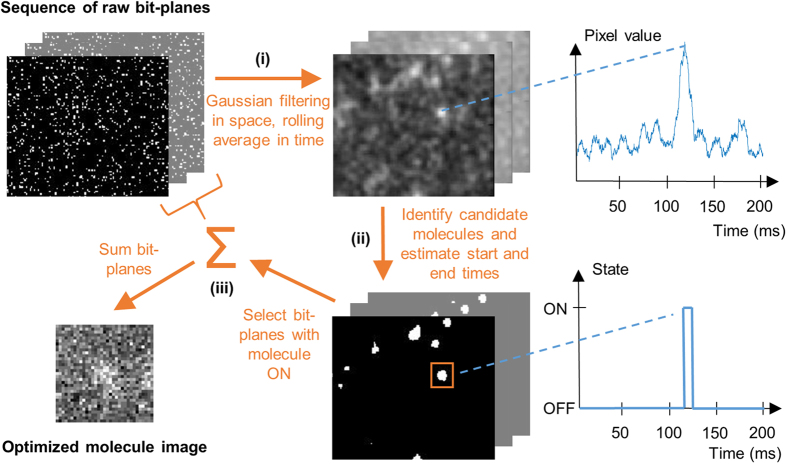
The smart-aggregation scheme. A sequence of bit-plane images are processed according to (i) filtering, (ii) molecule detection and positive photon events registration and (iii) summation of original bit planes according to aggregated photon arrival times.

The steps in the bit-plane smart aggregation algorithm are outlined in Fig. 2. The input to the system is the raw or unprocessed bit-plane images from the sensor. The first step is to apply a standard spatial Gaussian filtering, to enhance any molecule flashes^16^. The size (or width σ) of the Gaussian kernel is chosen so as to match the expected point spread function (PSF) of the molecule, readily estimated from the system optics. More specifically, *σ* is chosen to be one-third of the Airy disk radius, which is estimated from *r* = 0.61 *λ/NA*, where λ is wavelength and *NA* is the numerical aperture of the objective. The spatial filtering is followed by time averaging, carried out with a rolling “window”^17^, whose size corresponds to the shortest blink that can be reliably detected.

In the second step, thresholding is applied to the filtered frames to detect molecule flashes. Clusters of points are thereby identified as (potential) molecules, and in each case the local maximum of the filtered pixel values is used as a rough estimate {*x*_i_, *y*_i_} of the molecule position, which then defines a region of interest (ROI) around the candidate molecule. Next, the thresholded time trace at {*x*_i_, *y*_i_} is used to estimate the on and off time of the molecule flash in question.

The third step takes each candidate molecule in turn, selects the raw bit-planes deemed to contain the molecule flash (based on the estimated on and off times), and sums said bit-planes, cropped to the relevant ROI. The end result is a series of optimised images, which can then be readily localised using standard methods^18^. The overall scheme is highly parallelizable and thus well suited to implementation on FPGA.

The duration of the time averaging window, and the threshold used for detecting molecules are derived from the underlying photon detection statistics and are a function of the photon flux from the molecule, the background photon level (including the dark count rate), as well as the effective pixel size (or optical magnification).

To quantify the smart aggregation, consider a binary SPAD camera whose pixels have a uniform PDE *η*. For simplicity, each pixel is assumed to detect photons at the same average rate of *b* times per second when subjected to background light only. It is further assumed that a molecule, when on, emits a photon flux of *I* photons per second onto the sensor. The light from the molecule has a PSF centred around pixel {*i*, *j*}, and is approximated by a Gaussian with standard deviation *σ* (normalised by the pixel size). It is also assumed that bit-planes are captured by the camera with an exposure time of *τ* and are filtered spatially with a *k* × *k* Gaussian filter (also of width *σ*), before being summed, in time, in groups of *N*. It can then be shown, using the Poisson statistics of photon arrivals, that the mean value (E) of pixel {*m*, *n*} (as seen on the raw bit-planes) is:


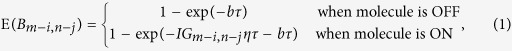


where *G* is the Gaussian PSF, as sampled (discretized) over the pixel array. Similarly, the variance (Var) of the pixels can be expressed as:


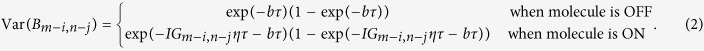


Now provided the pixel values *B* can be treated as independent random variables, which are uncorrelated in time (within an on or an off period), the pixel {*i*, *j*} in the filtered *F*, and aggregated image *H* will have the following mean and variance:


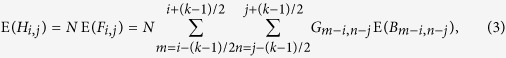



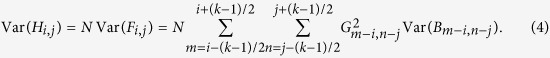


As *N* increases, and more and more pixel values, *B*, are summed to compose *H*, the distribution of *H* will tend to a Gaussian according to the Central Limit Theorem. The mean and variance of this distribution will depend on whether the molecule is on or off. Thus to ensure that the molecule can be reliably detected, the probability distributions in the on and off cases have to be suitably separated. If one is to aim for a ~99% detection accuracy based on the value of *H* at {*i*, *j*} (assuming the molecule was either on or off over the whole of the aggregation period), then the point where the tails of the two distributions intersect should be three standard deviations away from the means of the distributions, so the requirement is:


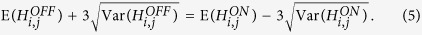


Thus, combining Eq. (5) with Eqs (3) and (4) one can show that the minimum number of bit-planes to be summed is:


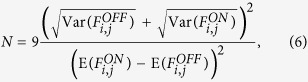


and the corresponding detection threshold *T,* which describes the acceptable lower detection limit of an on event above the average noise background, can be expressed as:


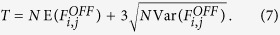


Now in practice *H* is a rolling sum of the filtered bit-planes *F*, so that at time step *t*,


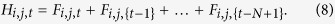


The precision with which the start and end times of the molecule flash can be determined will be dependent on the length of summation. One approach is to take the time corresponding to the middle of the sequence of *N* bit-planes when *T* is crossed (leading to an uncertainty in the estimated start and end times {*t*_s_, *t*_e_} of approximately ±*N*/2 bit-planes). When composing the optimised molecule image, the sum of bit-planes is instead carried out from *t*_s_ − *N*/2 to *t*_e_ + *N*/2 to reduce the chances of bit-planes with useful signal being discarded. To avoid a single molecule flash being treated as multiple flashes, instances of *H*_*i,j*_ dropping below *T* for a single frame only are ignored when establishing {*t*_s_, *t*_e_}.

A corollary of the above analysis is that the higher the anticipated photon flux from the molecule (or the PDE *η* the camera), the lower *N* can be, and hence the better one can establish the time history of the molecule flash (as well as being able to detect shorter on or off periods).

## Application of smart aggregation

To assess the benefit of smart aggregation, experimental studies were carried out in which the localisation uncertainty, obtained with the SPCImager, was compared with fixed aggregation and a reference emCCD camera. This was followed by further simulations involving a projected, future SPAD camera, which was compared with current emCCD and sCMOS devices to assess the future potential of smart aggregation bit-plane technology.

The statistical models used here to simulate the image capture process of the cameras are similar to those in the comparison study of ref. 19. In particular, photon detections and dark noise (in all three cameras) are assumed to be Poisson processes, the electron multiplication stage of the emCCD is modelled as a Gamma process, and read noise is Gaussian distributed. An important difference in the simulations here is that the molecule undergoes stochastic blinking so that the principle of smart aggregation may be tested.

For the simulations camera properties are defined as shown in Table 1. For the SPAD camera a 32 × 32 pixel region of interest (ROI) is assumed; for the emCCD and sCMOS camera models the ROI is 16 × 16 and 40 × 40, respectively (to ensure similar fields of view). The simulated molecules switch on, one at a time, at random intervals and locations, and with a given blink duration. As in the analysis described above, the molecule PSF is approximated by a Gaussian function.

### Experimental results

In the experiments, a sample of GATTA-PAINT 80 G nanorulers^20^ was captured using SPCImager and a Hamamatsu ImageEM emCCD camera. The sample consist of triplets of fluorescent markers, based on the ATTO 550 dye, that are 80 ± 5 nm apart, and is intended to be used as a calibration slide for super-resolution microscopy. Both cameras were coupled to an Olympus Cell Excellence IX81 microscope operated in a TIRF configuration, with a 561 nm excitation laser and a 150×, 1.45 NA, TIRF objective. A 50/50 non-polarizing beam splitter unit (TwinCam by Cairn Research) was used to direct the image onto each camera for simultaneous imaging.

In the first test, SPCImager and the emCCD device were used sequentially to image different fields of the GATTA-PAINT nanorulers. In the case of SPCImager, frames were generated by two different means: by summing non-overlapping groups of 640 bit-planes (to match, approximately, the emCCD’s frame time of 64 ms) and by using the smart aggregation scheme described previously to produce “optimised” molecule images, 32 × 32 pixels in size (the algorithm, implemented in Matlab, was applied to blocks of 10000 bit-planes at a time to limit RAM usage). For the smart aggregation the number of bit-planes summed, N, was 100 and the detection threshold, T, was 2. Background subtraction was carried out and hot pixels, defined as those giving a value of ‘1’ on more than 20% of background bit-planes, were interpolated over using the method of D’Errico^21^. The background and molecule intensity levels inferred from the camera outputs were fed into the simulation models and used to generate corresponding sets of simulated images. Both the experimental and simulated image frames were then localised using Maximum Likelihood fitting, via the widely-used ThunderSTORM ImageJ plugin^21^. The localisation errors obtained from the simulated data (for which the ground truth molecule positions are known) were compared with the localisation uncertainties reported by ThunderSTORM for the true emCCD and SPCIMAGER frames, as a means of validating the simulations models. ThunderSTORM employs the Thompson formula to estimate localisation uncertainty^14^. This assumes the dominant noise source is shot noise, directly applicable to SPAD arrays where background is a combination of DCR and background photons.

**Figure 3:**
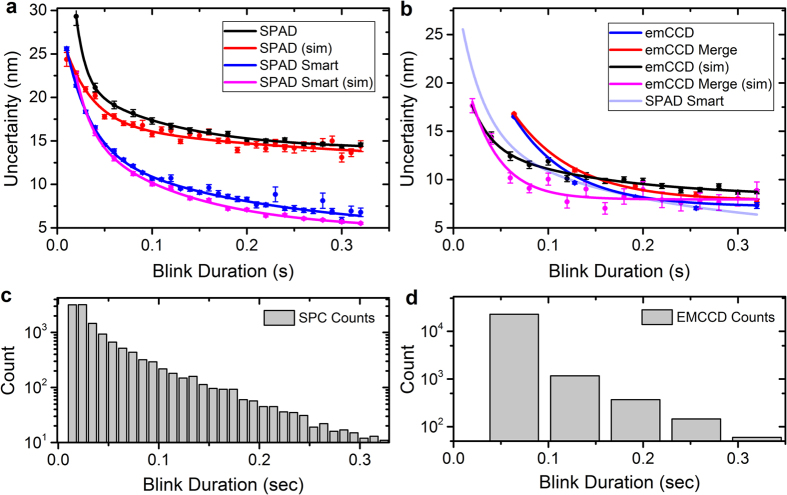
Single molecule localisation uncertainty for varying molecule blink durations as determined for (**a**) the SPAD SPCImager with and without smart-aggregation and (**b**) the emCCD with and without merged frames. Shown are both experimental data from GATTA-Paint 80 G nanorulers and simulated data. The solid lines are visual guides for the eye. (**c**) and (**d**) Shows histograms of estimated experimental blink duration of single molecules, as obtained from (**c**) SPCImager and (**d**) emCCD with 50 msec frame rate.

Figure 3a plots the simulated and experimental data for SPCImager (fixed and smart aggregation) and in Fig. 3b those from the emCCD. The vertical axes represent the root mean square (RMS) error in the localisation, or, in the case of the experimental data, the combined uncertainty (also a RMS quantity). The uncertainty and error values are plotted against a range of blink durations, as prescribed in the simulations, and determined approximately from the experiment data (based on SPCImager’s aggregation algorithm or the number of consecutive emCCD frames that the molecule is present such that the localisations are within 40 nm from one another on subsequent frames). The measured blink durations, in the case of SPCImager, are rounded to the nearest 10 ms so that experimental data points with similar blink durations may be combined and overall uncertainty figures calculated. Similarly, for the emCCD data, combined uncertainty values are computed, with data points being grouped according to the number of frames that the molecule is emitting. In addition, a separate set of uncertainty values are obtained, based on merging localisations deemed to result from the same molecule blink (to produce a comparable data set to SPCImager smart aggregation). Our emCCD read-out time is 30 ms and, as discussed above, therefore a proportion of the shorter blinks will invariably be missed.

The results in Fig. 3 show that for the SPCImager, smart aggregation of the bit-planes offers a clear improvement in localisation compared with fixed aggregation, with the uncertainty reducing by a factor of two for longer blink durations. For the emCCD, merging localisations also result in a reduction in uncertainty, though to a much lesser extent of around 20%. At short blink durations the improvement is reduced, largely due to the reduced photon numbers globally limiting localisation certainty. The simulated data matches well to the experimental data, providing confidence in the simulation. The small discrepancy between the simulations and experiment is largely due to the variability in the blink intensity being unmodelled in the simulations. Furthermore, ThunderSTORM’s uncertainty estimates (for the experimental data) do not take into account the non-uniformity in photon response and dark count across the SPAD array (and in the case of emCCD, the software does not account for read noise).

It is interesting to note in the data presented in Fig. 3, that SPCImager matches the performance of emCCD camera for long blink durations, despite the higher sensitivity of the latter device. The reason for this is that long blinks, spreading over multiple emCCD frames, result in several localisations, some of which can be imprecise due to reduced photon counts (when the molecule blink just spills into an extra frame) and may not be recognized as coming from the same molecule. The additional “poor” localisations lower the overall precision, though they can be avoided, to some extent, by increasing the detection threshold (or filtering localisations based on the estimated uncertainty). This problem does not arise for smart-aggregated bit-plane imaging. Figure 3c and d compare histograms of the blink duration, as estimated from the experimental data from the two cameras (via the smart aggregation scheme, and counting the number of consecutive emCCD frames showing the same apparent blink). The two histograms are similar in shape, though SPCImager provides higher time resolution, allowing the decay in the apparent distribution of blink durations to be observed in more detail.

To verify the localisation accuracy and precision, SPCImager and the emCCD device captured the same field of view of a GATTA-PAINT nanoruler sample simultaneously for 20 minutes using the TwinCam 50/50 unit. To accommodate the lower signal to noise ratio resulting from splitting the light into two, the Thunderstorm molecule detection thresholds were raised from the default value (as detailed in Table 2). Moreover, the thresholding of SPCImager images was modified on account of the non-uniform noise affecting the sensor array. Beyond these two factors, the fitting parameters are identical between the two cameras. For smart aggregation, the number of summed images, N, was 400, and the threshold, T, 6, higher values from Fig. 3 due to the lower absolute photon flux the 50/50 splitter introduces.

**Figure 4:**
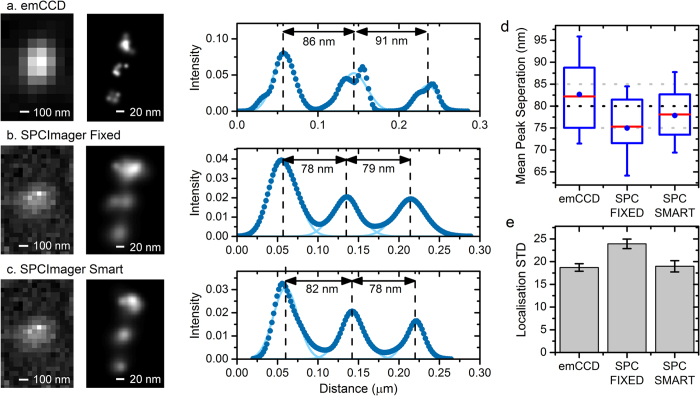
Single Molecule STORM localisations of a single GATTA-PAINT 80 G nanoruler as captured by emCCD and SPCImager, analysed as both standard and smart aggregation. (**a**–**c**) Shows, from left to right, the image of the single nanoruler pre-localisation, the localised nanorulers and the cross section through the localisations. For the smart aggregation analysis, N = 400 and T = 6. (**d**) and (**e**) Show the localisation distances and localisation STD of the nanoruler localisation for 5 different nanorulers (15 markers).

Figure 4a–c shows an example of the same nanoruler, as localised from emCCD, SPCImager fixed frame (matching emCDD frame time of 120 ms), and SPCImager smart frames. The localisations are visualised using the normalised Gaussian option of ThunderSTORM (left plots), whereby each localisation contributes to the rendered image in the form of a Gaussian function with standard deviation equal to the estimated uncertainty. ThunderSTORM’s density filter has been applied to filter out localisations with fewer than three neighbours within a 30 nm radius, so as to remove isolated localisations resulting from noise or autofluorescence. Note that as each constituent marker within the nanoruler blinked multiple times during the imaging period, every marker is reproduced as a scatter of localisations, with the scatter relating to the density of localisations. The right plots show the cross-section of the Gaussian-rendered localisation map of the triplet Gatta system and Fig. 4d shows the statistics of the mean localisations for the markers of five nanorulers. Peak-to-peak distances map well with the calibrated 80 nm GATTA nanoruler sample, within the ±5 nm error, for both cameras and all analysis techniques,

Figure 4e plots the standard deviation of the localisations for the markers in Fig. 4d. The emCCD and SPC Imager with smart aggregation show similar results, with both returning significantly better localisations than the SPCImager without smart aggregation. This is consistent with the results of Fig. 3b, where we compare the data from Fig. 4 with an overall average of all blink durations from Fig. 3. This shows that, by taking advantage of the faster frame rate and dark count noise floor, smart aggregation returns competitive results from the SPCImager when compared to a commercial emCCD, despite the 4x less fill factor and 2.5x less quantum efficiency. This provides confidence that a new generations of SPAD array cameras will outperform existing emCCD’s for SMLM.

### Projections for future SPAD device

To explore and assess the future potential of a SPAD imager, simulations have been performed assuming an ideal, experimentally realistic, SPAD array (F-SPAD). It was assumed that the effective fill factor will, in time, increase three-fold, with the DCR reducing by a factor of four. It is believed that these are realistic assumptions; there are a number of avenues in which such a fill factor improvement may be realized, including microlensing^22^, back-side illumination^23^ or stacking^24^. Moreover, experimental studies suggest that SPADs with a similar structure to the sensor studied here exhibit a halving of DCR for every 8 °C of cooling until around −10 °C^25^. Thus it is anticipated that a four times reduction in DCR is achievable with a moderate level of cooling.

As before, the simulations considered a range of molecule blink durations, and for each duration 500 images were generated, localised, and the individual localisation errors (distances to ground truth) combined to form an overall mean square error. Localisation was again performed using ThunderSTORM on the simulated images. Localisations further than 53.3 nm from the ground truth (corresponding to the effective pixel size with a 150× objective) were considered to be false detections. The photon flux from the molecule was taken to be 50000 photons/sec, with a background level of 70000 photons/sec for every μm^2^ area in the sample (under the assumption of TIRF conditions). Furthermore, the microscope was assumed to feature a 150× (in the default case) or a 60x objective. An overview of camera characteristics and details of the cameras being modelled in the simulations, is given in Table 1.

**Figure 5:**
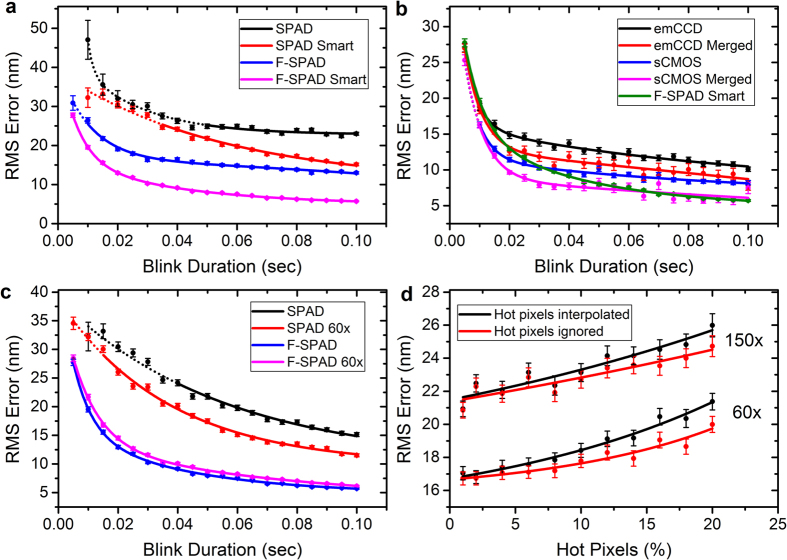
Projections from model simulations. (**a**) Localisation performance of current SPAD versus a modelled future SPAD (**b**) Future SPAD as compared with sCMOS and emCCD. (**c**) The effect of 60x objective on the localisation of current and future SPADs and (**d**) the effect of hot pixels on the localisation error of current SPAD. All solid lines are guides to the eye. Dashed lines represent blink durations with <90% detection sensitivity.

Figure 5a compares the localisation error resulting from the current SPCImager, with the projected, F-SPAD. Both fixed and smart frame aggregation are considered. The error is seen to (approximately) half with the future SPAD device. Figure 5b shows the F-SPAD (with smart aggregation) as compared with existing commercial emCCD and sCMOS cameras. As previously, we consider the effect of merging consecutive emCCD/sCMOS localisations if within 40 nm of each other. The solid lines are guides to the eye, with the dashed section showing blink duration events with <90% detection sensitivity. The results show the future SPAD camera outperforming the emCCD for almost all blink durations. The F-SPAD matches the sCMOS in all but below the 20–30 ms times, in which case sCMOS gives better localisation results, but lower detection sensitivity, dropping below the 90% threshold. The SPAD camera remains above 90% for all blink durations. Figure 5c shows the effects of switching to the 60x objective, for the cases of the current and future SPAD camera and smart aggregation is assumed throughout. It is noted that the 60x objective reduces the localisation error of the current SPAD by a moderate extent (by around 20% for longer blink durations), but has negligible effect on the F-SPAD. The reason is that with the future camera, the DCR is no longer significant; the error is mainly caused by the background photon count, which simply gets re-distributed across the pixel array as the magnification is changed (in a similar way, the simulated error curve for the sCMOS was found to be largely unaffected by the change in assumed objective).

Figure 5d considers the effect of an increasing number of hot pixels in a SPAD camera. Hot pixels have thus far not been modelled here, but their percentage can be considerable in an uncooled SPAD (in the region of 10%). For this simulation, the model for the current SPAD was used with 50 ms total exposure and the molecule being on throughout. The simulated images were localised using the Maximum Likelihood Estimator code of Smith *et al*.^26^, which was modified to allow for undefined pixel values. The graph plots the results of two different strategies for handling hot pixels: interpolating over them (using the method of ref. 20, as applied to the experimental data in Section 4.1) and ignoring hot pixels altogether. Both 60x and 150x objectives are considered. In both cases, ignoring hot pixels appears to be the preferable option, but the significant result is that even with 20% hot pixels, there is only a small increase in localisation error, and hardly any change with fewer than around 10% hot pixels. This result is significant as it suggests that hot pixel removal is not a priority for applications to SMLM.

## Conclusion

Binary SPAD cameras, operating at a high frame rate, offer flexibility in the imaging of blinking molecules. A technique, termed “smart aggregation” has been presented here, which, for every detected molecule, sums only those binary fields when the molecule is on, for optimised images. The advantage of the approach has been demonstrated in both simulations and experiments involving nanorulers, with localisation errors reducing considerably. The simulations suggest that with the anticipated improvement in fill factor and reduction in dark noise, a future SPAD imager featuring smart aggregation will match or outperform existing sCMOS and emCCD cameras in molecule localisation.

Whilst the present work uses standard localisation algorithms, a camera-specific scheme (as in ref. 27 for sCMOS cameras), which takes into account the non-uniformity in the pixel array, would likely perform better. It is expected that such as scheme, when applied to SPAD data, would result in higher accuracy, and a more symmetric distribution of localisations around the ground truth.

## Additional Information

**How to cite this article**: Gyongy, I. *et al*. Smart-aggregation imaging for single molecule localisation with SPAD cameras. *Sci. Rep.* **6**, 37349; doi: 10.1038/srep37349 (2016).

**Publisher's note:** Springer Nature remains neutral with regard to jurisdictional claims in published maps and institutional affiliations.

## Figures and Tables

**Table 1:** Details of the camera specification as used in the model.

Type	Binary SPAD	emCCD	sCMOS
Model	SPCImager	Andor iXon Ultra 987	Hamamatsu ORCA-Flash4.0 V2
Pixel size	8 μm	16 μm	6.5 μm
Quantum Efficiency	35% PDE	90%	80%
Fill factor	26% (78%)	N/A	N/A
Read noise	Negligible	0.2 e^-^ (input referred)	1.4 e^-^
Dark Noise	100 Hz (25 Hz) median DCR	0.001 Hz DCR	0.05 Hz mean DCR
Other Noise	N/A	0.0018 Hz CIC	N/A
Non-uniformity	2% DRNU, 1% PRNU	N/A	1% DRNU, 0.5% PRNU
Frame time	100 μs per bit-plane	50 ms	50 ms

**Table 2:** ThunderSTORM molecule detection settings for simultaneous emCCD/SPCImager imaging.

Process	emCCD	SPCImager
Image filtering (default: Wavelet filter)	Wavelet filter	Gaussian filter (sigma = 2 pixels)
Peak intensity threshold (default: std(Wave.F1))	4*std (Wave.F1)	mean (Med.F) + std (Wave.F1)

Wave.F1 is the first wavelet level of the input image, and Med.F is the median filtered input image.
